# Extensive tissue-specific transcriptomic plasticity in maize primary roots upon water deficit

**DOI:** 10.1093/jxb/erv453

**Published:** 2015-10-13

**Authors:** Nina Opitz, Caroline Marcon, Anja Paschold, Waqas Ahmed Malik, Andrew Lithio, Ronny Brandt, Hans-Peter Piepho, Dan Nettleton, Frank Hochholdinger

**Affiliations:** ^1^Institute of Crop Science and Resource Conservation, Crop Functional Genomics, University of Bonn, D-53113 Bonn, Germany; ^2^Institute for Crop Science, Biostatistics Unit, University of Hohenheim, D-70599 Stuttgart, Germany; ^3^Department of Statistics, Iowa State University, Ames, IA 50011-1210, USA; ^4^Leibniz Institute of Plant Genetics and Crop Plant Research, D-06466 Gatersleben, Germany

**Keywords:** Drought, expression pattern, low water potential, maize, primary root, RNA-Sequencing (RNA-Seq), tissue specificity, transcriptome, water deficit.

## Abstract

Water deficit is the most important environmental constraint severely limiting global crop growth and productivity. This study investigated early transcriptome changes in maize (*Zea mays* L.) primary root tissues in response to moderate water deficit conditions by RNA-Sequencing. Differential gene expression analyses revealed a high degree of plasticity of the water deficit response. The activity status of genes (active/inactive) was determined by a Bayesian hierarchical model. In total, 70% of expressed genes were constitutively active in all tissues. In contrast, <3% (50 genes) of water deficit-responsive genes (1915) were consistently regulated in all tissues, while >75% (1501 genes) were specifically regulated in a single root tissue. Water deficit-responsive genes were most numerous in the cortex of the mature root zone and in the elongation zone. The most prominent functional categories among differentially expressed genes in all tissues were ‘transcriptional regulation’ and ‘hormone metabolism’, indicating global reprogramming of cellular metabolism as an adaptation to water deficit. Additionally, the most significant transcriptomic changes in the root tip were associated with cell wall reorganization, leading to continued root growth despite water deficit conditions. This study provides insight into tissue-specific water deficit responses and will be a resource for future genetic analyses and breeding strategies to develop more drought-tolerant maize cultivars.

## Highlight

Maize primary root tissues display extensive transcriptomic plasticity upon water deficit. The most significant adaptive changes in the elongation zone lead to reprogramming of metabolism and cell wall organization.

## Introduction

In agriculture, drought is responsible for more yield losses than any other abiotic stress (Boyer, 1982). Besides widespread poor soil moisture, variations in water availability within fields result in uneven crop stands and cause yield losses (Nafziger *et al.*, 1991). As a consequence of global warming, land areas subjected to drought conditions increase each year. Thus, new insights into the molecular mechanisms of drought response and adaptation are required to advance breeding and enable an estimated 70% increase in crop production until 2050 to feed the growing world population (Tester and Langridge, 2010).

When plants experience drought conditions, their growth is inhibited. Root development is less sensitive to water deficit than shoot development or leaf growth (Westgate and Boyer, 1985; Sharp *et al.*, 1988). Root growth maintenance is an important adaptive trait ensuring that plants can access deep water and nutrient resources to survive (Rodrigues *et al.*, 1995). It relies predominantly on the activity of the plant hormone abscisic acid (ABA). ABA accumulation prevents excessive ethylene production and growth inhibition. Furthermore, ABA promotes transport of proline to the root apex and thereby contributes to osmotic adjustment. Hence, cellular turgor is protected and a favourable water potential gradient towards the environment is maintained; that is, a more negative water potential than in the outside medium, enabling water influx. Maintenance of root elongation further depends on an increase of longitudinal cell wall extensibility (reviewed in Yamaguchi and Sharp, 2010). Other drought stress responses limit cellular damage by sustaining protein structure, maintaining membrane integrity, and removing toxic compounds such as reactive oxygen species (ROS) to restore redox balance (reviewed in Krasensky and Jonak, 2012). In spite of the well-known physiological processes, relatively little is known about the underlying gene regulatory networks that translate environmental changes to metabolic alterations needed to gain stress tolerance (Spollen *et al.*, 2008; Krasensky and Jonak, 2012).

Maize is one of the most important economic crops worldwide and an important source for food, fodder, and energy. Due to climate change, drought is a growing threat to most maize cultivation areas (IPCC, 2014). However, breeding approaches have not yet decreased maize drought sensitivity (Lobell *et al.*, 2014), making it important to understand the molecular mechanisms underlying the drought response.

Global gene expression profiles in response to water deficit have been monitored in different parts of maize including roots, leaves, and kernels by microarray chip hybridization experiments (Zheng *et al.*, 2004; Andjelkovic and Thompson, 2006; Jia *et al.*, 2006; Zhuang *et al.*, 2007; Yue *et al.*, 2008; Hayano-Kanashiro *et al.*, 2009; Marino *et al.*, 2009; Luo *et al.*, 2010; Downs *et al.*, 2013; Humbert *et al.*, 2013) and more recently by RNA-Sequencing (RNA-Seq) (Kakumanu *et al.*, 2012; Opitz *et al.*, 2014). RNA-Seq allows for fully quantitative gene expression analyses with absolute values and the capture of even subtle expression changes. These studies provided global insight into the maize water deficit response; however, the composite nature of the studied organs limited their spatial resolution. Plant organs are made up of different cell and tissue types each featuring a unique transcriptome (Brady *et al.*, 2007). Hence, transcriptome analyses of entire organs yield average gene expression profiles integrated over all cell types, thus potentially masking genes of interest (Schnable *et al.*, 2004). To date, there are only few transcriptome studies addressing this problem in maize roots focusing on different parts of the elongation zone after drought stress treatment (Bassani *et al.*, 2004; Sharp *et al.*, 2004; Poroyko *et al.*, 2007; Spollen *et al.*, 2008). Plant roots can be divided into specialized zones of development, including from the terminal end the root cap, the apical meristem, the cell elongation zone, and the maturation zone (Ishikawa and Evans, 1995). The root cap covers and thus protects the root tip, secretes mucilage to facilitate root movement, and acts as a sensor for gravity, light, temperature, and moisture gradients. The root apical meristem forms new cells that start to elongate and move proximally into the elongation zone where elongation reaches a maximum. The elongation zone is critical for the reaction to exogenous signals including the root growth response to water deficit. Eventually, cells dislocate into the maturation or differentiation zone and acquire their distinct functions (Ishikawa and Evans, 1995). The differentiation zone depicts in radial orientation several functionally diverse cell types. As an outermost layer, an epidermis that consists of root hair-forming trichoblasts and non-root hair-forming atrichoblasts encloses the root. The epidermis takes up water and nutrients, which are then either transported into aboveground plant organs or metabolized within the root. The epidermis is followed by multiple layers of cortex tissue and a single layer of endodermis. The adjacent pericycle represents the outermost layer of the central cylinder or stele which contains alternating poles of differentiated xylem vessels functioning in water and nutrient transport, and primary phloem elements instrumental in transport of photosynthates. The vasculature elements are embedded by parenchymal pith tissue that forms the centre of the root (Hochholdinger, 2009).

Polyethylene glycol (PEG) treatment was demonstrated to simulate the occurrence of drought stress in drying soil (Zheng *et al.*, 2004). In the present study, maize seedlings were exposed for 6h to PEG8000 solution with a water potential of –0.8MPa. Thus, water availability was in the mid range of naturally occurring, plant-usable soil water potentials, representing mild water deficit conditions (O’Geen, 2012). From an agronomic viewpoint, such moderate drought conditions are of great importance as they are frequent and reduce yield (Sinclair, 2011). During long-term (>24h) exposure to such a low water potential, maize primary root growth is reduced by 30–50% (Saab *et al.*, 1992; Sharp *et al.*, 2004; Opitz *et al.*, 2014). However, short-term treatment did not impair root growth (Opitz *et al.*, 2014) and can therefore be used to monitor gene expression changes that precede the physiological changes. After treatment, the meristematic zone, the elongation zone, and the cortex and stele of the differentiation zone were mechanically separated and their transcriptomes analysed by RNA-Seq. The main goal of this study was to explore the tissue-specific plasticity of the transcriptomic landscape in response to water deficit.

## Materials and methods

### Plant material and water deficit treatment

Surface-sterilized seeds of the maize inbred line B73 were germinated in paper rolls as previously described (Ludwig *et al.*, 2013). After 4 d, seedlings with a primary root length of 2–4cm were transferred to new paper rolls saturated with PEG8000 (*M*_r_ 7300–9000Da; Roth, Karlsruhe, Germany) solution with a water potential of –0.8MPa for water deficit treatment, or distilled water for control experiments, as previously described (Opitz *et al.*, 2014). After 6h of incubation in aerated PEG solution or distilled water, primary roots were dissected into four distinct tissues. First, the apical 2mm of the root were collected, containing the root cap and meristematic zone. Secondly, the proximal zone adjacent to the root tip up to the part of the root where the root hairs became visible was sampled, which corresponds to the elongation zone. Finally, the distal differentiation zone, from the root hair zone to the coleorhiza, was mechanically separated into cortex (including epidermis, cortical parenchyma, and endodermis) and stele (including pericycle, phloem, xylem, and pith parenchyma) according to Saleem *et al.* (2010). Briefly, after cutting the apical tissues off, the root was incised with a razor blade close to the coleorhiza. The cortex was then pulled off the stele by gently bending the root while the stele remained attached to the remainder of the seedling. Collected plant material was immediately frozen in liquid nitrogen and stored at –80 °C until RNA isolation. Experiments were performed in four biological replicates each consisting of 10 pooled root tissues from the same 10 seedlings.

### RNA isolation and sequencing library preparation

Pooled primary root tissues were ground in liquid nitrogen, and total RNA was isolated with the RNeasy Plus Universal Mini Kit (Qiagen, Venlo, The Netherlands). RNA quality was assessed via agarose gel electrophoresis and a Bioanalyzer (Agilent RNA 6000 Nano Chip; Agilent Technologies, Santa Clara, CA, USA). For all samples, a RIN (RNA integrity number) ≥8.5 was detected. cDNA libraries for Illumina sequencing were constructed according to the protocol of the manufacturer (TruSeq RNA Sample Preparation; Illumina, San Diego CA, USA). For sequencing, each library was indexed by an Illumina TruSeq Adapter in which no adaptor was used more than twice (Supplementary Table S1 available at *JXB* online). Indexed libraries were loaded onto a flow cell following an incomplete block design (generated with CycDesigN 5.1) with four pooled libraries per lane, which was considered as an incomplete block (Supplementary Table S1). Cluster preparation and single read sequencing were performed according to the manufacturer’s instructions (HiSeq 2000, Illumina).

### Processing and mapping of sequencing reads

Raw sequencing reads were processed and subsequently mapped with CLC Genomics Workbench (Version 7.0; http://www.clcbio.com/products/clc-genomics-workbench/). Reads with more than one mismatch in the adapter sequence were excluded. Quality trimming removed low quality and ambiguous nucleotides of sequence ends and adapter contamination. Only reads ≥40bp were retained for further analyses. These were initially mapped to the maize B73 reference genome (RefGen_v2; ftp://ftp.gramene.org/pub/gramene/maizesequence.org/release-5b/assembly/; Schnable *et al.*, 2009) allowing large gaps of up to 50kb to span introns. To be mapped, at least 90% of each read had to fit with 90% similarity to the reference. Stacked reads (i.e. redundant reads sharing the same start and end co-ordinate), sequencing direction, and sequence were merged into one. The remaining reads were further projected to the ‘filtered gene set’ (FGS_v2; ftp://ftp.gramene.org/pub/gramene/maizesequence.org/release-5b/filtered-set/; Release 5b) of the B73 reference genome. Thereby, reads had to fit at least with 80% of their length comprising 90% similarity to the reference. Only reads uniquely mapping to the reference were further analysed.

Sample relationships were displayed and compared in a hierarchical clustering, and a multidimensional scaling plot was produced with the Bioconductor package ‘edgeR’ in R. The multidimensional scaling plot displays the distances between the samples as the leading-log_2_ fold change corresponding to the estimated root-mean-square deviation of the top genes with the largest standard deviations between samples.

### Statistical procedures to determine active and inactive genes

A generalized linear mixed model with a negative binomial response was used to model gene-wise transcriptional activity for each tissue–treatment combination. Conditional on random effects for sequencing lanes and biological replicates, the log of the mean read count was assumed to be the sum of a normalization factor and a linear function of the random and fixed effects for each combination of tissue and treatment. Each normalization factor was calculated by adding the log of the TMM normalization factor (Robinson and Oshlack, 2010) for each sample to the fitted value of a smoother on the log of the mean of the raw counts using GC content and gene length as covariates.

The vector of fixed effects for each gene was assumed to be a draw from a multivariate normal distribution with an unknown and unrestricted mean and an unknown diagonal variance–covariance matrix. The log of the negative binomial dispersion parameter was assumed to be constant within a gene, and a draw from a normal distribution with unknown mean and variance. The precisions of the random effects were assumed to follow gamma distributions, where the parameters for the lane effects were specified to create a vague distribution. The unknown parameters in the distributions of the fixed effects, negative binomial dispersion, and precision of the biological replicate effects were estimated using an empirical Bayes procedure via the R package ‘ShrinkBayes’ (Van De Wiel *et al.*, 2012). Integrated nested Laplace approximation (Rue *et al.*, 2009) was used to approximate the posterior distribution for the fixed effect associated with gene, tissue, and condition.

For a given threshold T, the posterior distribution for gene g, tissue t, and condition c was used to find P_gtc_(T), which is the posterior probability that the fixed effect for gene g, tissue t, and condition c was larger than T. Gene g was called ‘active’ for tissue t and condition c if P_gtc_(T) >0.5 and ‘inactive’ otherwise. Because fixed effects were estimated while accounting for sequencing differences from sample to sample, gene length, and GC content differences, classifying genes as active or inactive based on the posterior distribution of fixed effects is more meaningful than attempting to do so based on a single raw read count threshold applied to all genes. In terms of counts, the selected threshold T resulted in calling a gene of average length and average GC content active if the expected number of reads per million mapped reads was approximately ≥2. The read count threshold was, in effect, adjusted up or down for other genes depending on gene length, GC content, and the observed empirical distribution between these variables and read count.

### Statistical procedures for analysing differential gene expression

For differential expression analyses, read counts (+0.5) were log-transformed and mean variance trends estimated. Only genes with a minimum of five mapped reads in all four replicates of at least one tissue sample were considered. Based on the predicted variance, weights were assigned to each observation to adjust for heteroscedasticity in the linear modelling process (Law *et al.*, 2014). To borrow strength across genes in the estimation of the residual error variance, the empirical Bayes approach implemented in the Bioconductor package ‘limma’ (Smyth, 2005) was used in R. The data were further analysed by fitting a linear mixed model that included a lane effect, where lanes corresponded to the incomplete blocks of the experimental design. The lane effect was considered as a random effect, thus allowing the recovery of the interblock information. Four pairwise comparisons were computed, comparing water deficit with control treatment for each tissue. Resulting *P*-values of contrasts were corrected for multiplicity using the false discovery rate (FDR) approach of Benjamini and Hochberg (1995).

### Gene Ontology (GO) and metabolic pathway analyses

The web-based agriGO software (http://bioinfo.cau.edu.cn/agriGO/index.php) was used to assign GO functional categories to differentially expressed genes. Singular enrichment analysis computed over-represented categories in the sets of differentially expressed genes by comparing them with GO terms in the set of all expressed genes using Fisher’s exact test (Du *et al.*, 2010). Multiple testing was corrected by FDR (Benjamini and Yekutieli, 2001), and a cut-off was introduced at 5%.

The Mapman software (http://mapman.gabipd.org/web/guest/mapman; Thimm *et al.*, 2004) assigned differentially expressed genes to metabolic pathways and subsequently visualized them based on the functional annotation file ZmB73_5b_FGS_cds_2011. A χ^2^ test was used to determine if more genes than expected from the distribution of all expressed genes were assigned to each of the 32 major Mapman categories. The same test was used to evaluate the distribution of differentially expressed transcription factors (TFs) into 55 TF families (according to the Plant Transcription Factor Database v3.0; http://planttfdb.cbi.pku.edu.cn/; Jin *et al.*, 2013).

## Results

### RNA-Sequencing and mapping of root tissue transcriptomes

**Fig. 1.**
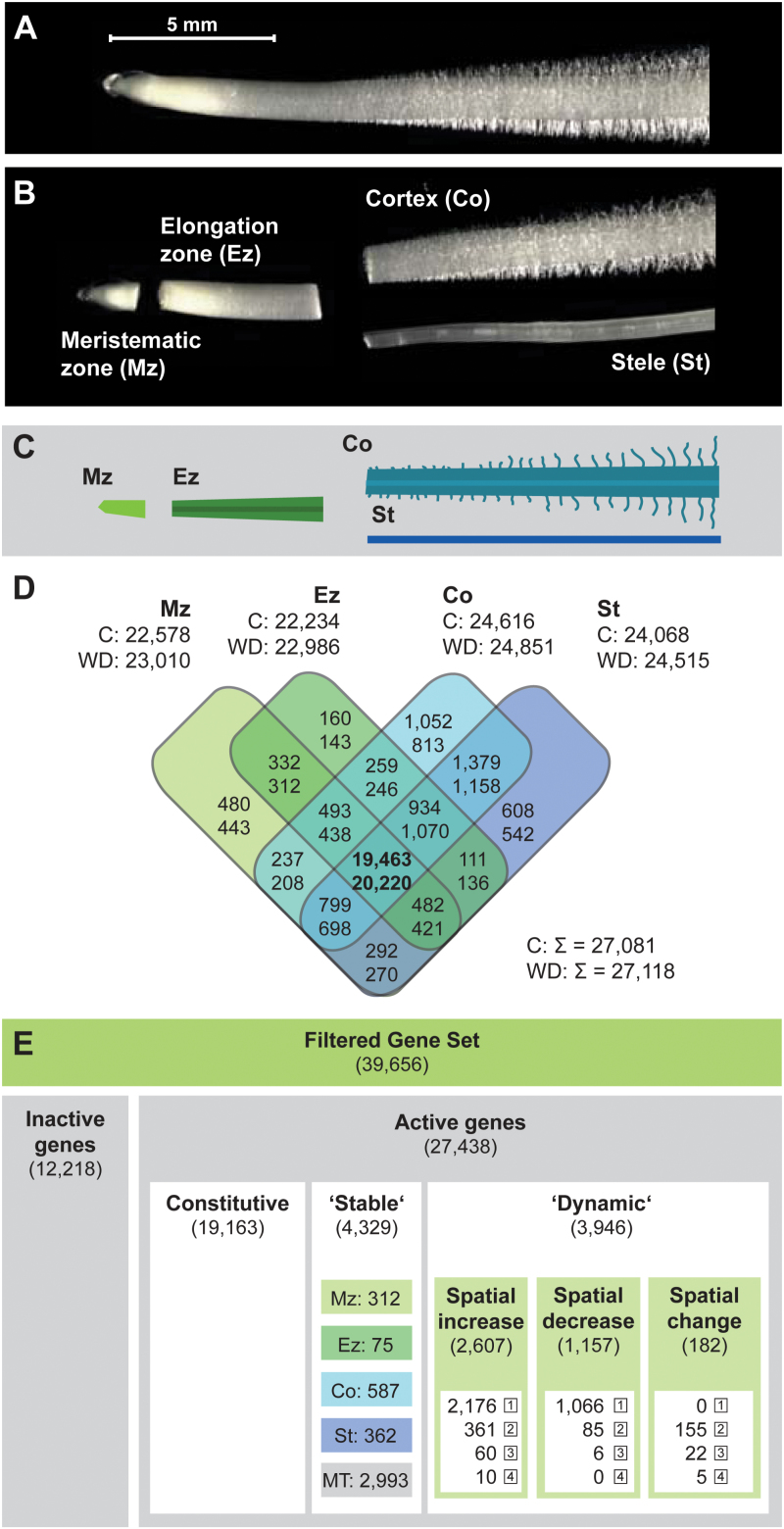
Maize primary root tissues and tissue-specific gene activity. (A) Maize primary root prior to tissue sampling. (B) Manually dissected root with separated meristematic zone, elongation zone, cortex, and stele. (C) Colour-coded schematic of dissected root. (D) Overlap of active genes under control (upper numbers) or water deficit conditions (lower numbers) in each tissue. (E) Subdivision of genes according to their activity status (see the text). Stable genes were active in the same tissue(s) under both conditions (activity in single tissues: Mz, Ez, Co, St; activity in multiple tissues: summed in MT). Dynamic genes were active in additional tissues (spatial increase), fewer tissues (spatial decrease), or different tissues (spatial change) upon water deficit. Numbers in boxes depict the number of activity status changes. Mz, meristematic zone; Ez, elongation zone; Co, cortex; St, stele; C, control; WD, water deficit treatment.

To survey the transcriptomic dynamics of maize root tissues in response to water deficit, seedlings were subjected to a low water potential of –0.8MPa and control conditions for 6h (see the Materials and methods). Subsequently, four root tissues were sampled, namely the meristematic zone comprising the apical meristem and root cap (Mz), the elongation zone (Ez), and both the cortex (Co) and stele (St) of the differentiation zone (Fig. 1A–C). For transcriptome sequencing, RNA was extracted in four biological replicates per tissue and treatment and converted into cDNA libraries for sequencing procedures. RNA-Seq yielded on average ~49 million 100bp reads per sample. The sequencing data have been deposited in the NCBI sequencing read archive (SRA; http://www.ncbi.nlm.nih.gov/sra; accession no. SRP052697). On average, 85% of high-quality reads mapped uniquely to the maize reference genome (Supplementary Table S1 at *JXB* online). After removal of duplicated reads, 74% of the remaining reads mapped to unique positions in the ‘filtered gene set’ (FGS; Supplementary Table S1), a set of 39 656 high confidence gene models.

### Gene activity under control and water deficit conditions

Gene-wise transcriptional activity for each tissue–treatment combination was computed. To decide if a gene was expressed (‘active’) in any tissue–treatment combination or not (‘inactive’) (Supplementary Table S2; Supplementary Fig. S1 at *JXB* online), a generalized linear mixed model was applied (see the Materials and methods). In total, 27 438 genes (69% of the FGS; Fig. 1E) were declared active. In the surveyed tissues, between 235 and 752 more genes were active after water deficit treatment (Fig. 1D). A substantial number of genes was constitutively active in all four tissues under control (19 463: 72%) and water deficit (20 220: 75%) conditions. In contrast, only 2300 (8%) and 1914 (7%) genes were specifically active in a single tissue (Fig. 1D). The majority of active genes (19 163: 70%) were constitutively active in all tissues under both conditions (Fig. 1E). The activity of the remaining genes was classified either as ‘stable’ or as ‘dynamic’. Stable genes were detected in the same tissues under both conditions and thus did not change their activity status; that is, they were neither turned on nor turned off in any tissue following treatment (4329 genes). A total of 31% of stable genes (1336/4329) were active in a single tissue, while 69% (2993/4329) were active in multiple tissues (Fig. 1E; Supplementary Fig. S1). On the other hand, 3946 genes were classified as dynamic genes. These genes changed their activity status in at least one tissue following water deficit treatment. Functionally, genes with stable and dynamic expression were related to similar metabolic pathways. However, genes involved in stress response processes were more numerous among the dynamic genes. Genes with dynamic activity were further categorized according to the type of change: the activity of 2607 genes spatially expanded and included more tissues after treatment, while the activity of 1157 genes was restricted to fewer tissues. A minority of 182 genes displayed an activity change in both directions (i.e. included different tissues after water deficit; Fig. 1E; Supplementary Fig. S1). Spatial activity changes were quantified; for example, a single change indicated a gene that was active in one tissue under control conditions and in the same tissue plus in an additional tissue after treatment. The maximum of four changes indicated a gene that was inactive under control conditions but active in all tissues after treatment (and vice versa). Ten genes displayed this kind of pattern, encoding HSF (heat shock factor) TFs 8, 17, and 20, TCP TF 36, a putative ABA signalling protein, two putative LEA (late embryogenesis abundant) proteins, and three unknown proteins. Overall, single changes were most common, while multiple changes were less frequent the more changes they included (Fig. 1E).

### Tissue-specific gene regulation in response to water deficit

**Fig. 2.**
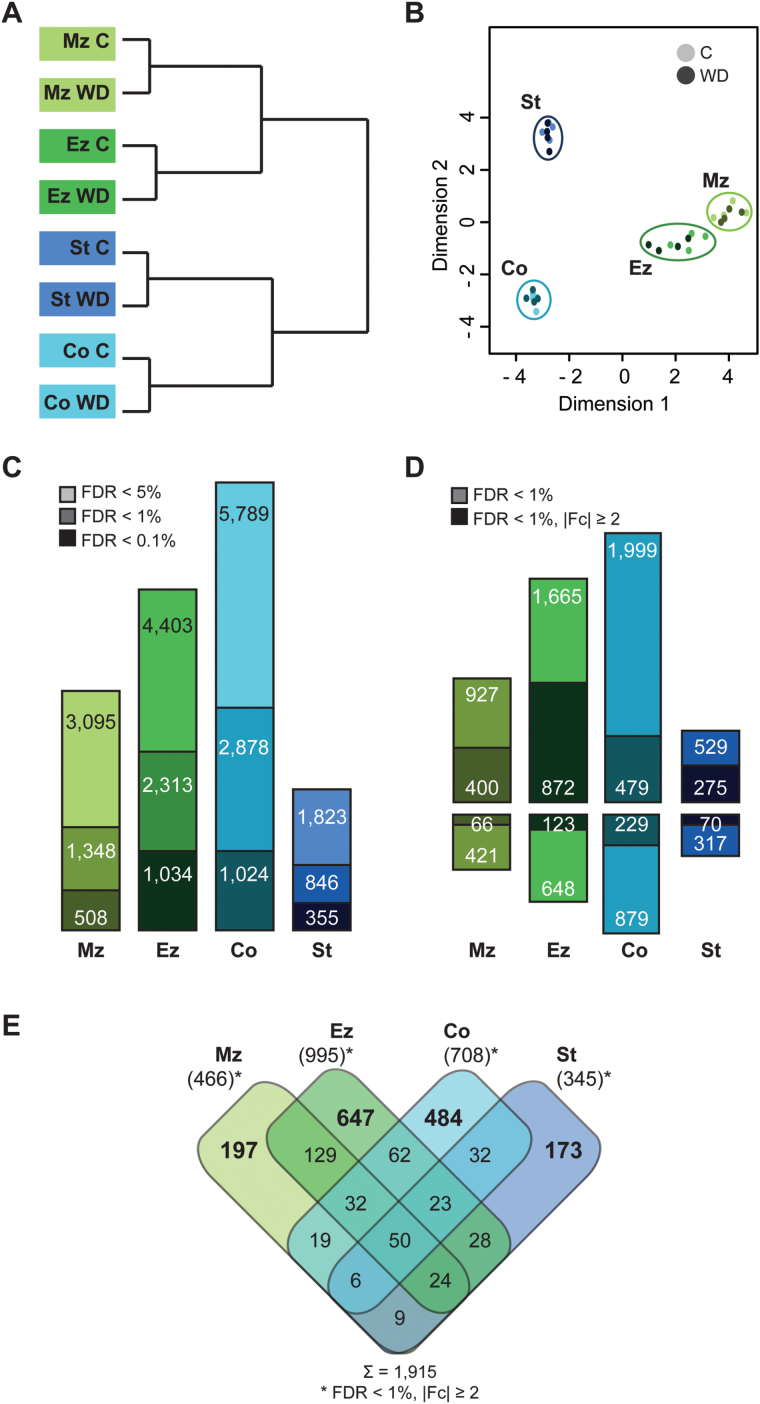
Sample relationship and differential gene expression. (A) Hierarchical clustering and (B) multidimensional scaling plot of root tissue transcriptomes. (C) Numbers of differentially expressed genes in the four root tissues at different significance levels (light colour, FDR <5%; medium dark colour, FDR <1%; dark colour, FDR <0.1%). (D) Numbers of up- and down-regulated genes (FDR <1%); dark bars represent strongly regulated genes (|log_2_Fc| ≥1). (E) Overlap between differentially expressed genes (FDR <1%; |log_2_Fc| ≥1) in the four root tissues. Mz, meristematic zone; Ez, elongation zone; Co, cortex; St, stele; C, control; WD, water deficit treatment.

Hierarchical clustering of sample data revealed that the transcriptomes of each root tissue clustered together under control and water deficit conditions (Fig. 2A). Therefore, differences between tissues were more severe than those between treatments. Additionally, the young, apical tissues of the meristematic and elongation zone and the mature tissues of the differentiation zone stele and cortex formed separate clusters (Fig. 2A). In a multidimensional scaling plot, the samples of the meristematic and elongation zone clustered closely together, while cortex and stele samples were more distantly related (Fig. 2B). To identify genes differentially regulated in response to water deficit treatment, pairwise contrasts between control and water deficit conditions were computed for each tissue, yielding four sets of differentially expressed genes (Fig. 2C; for a complete gene list including normalized expression values, log_2_ fold changes (Fc), and *q*-values, see Supplementary Table S3 at *JXB* online). The pattern of responsive gene quantities was cortex >elongation zone >meristematic zone >stele (Fig. 2C). Thereby, 2878 genes were differentially regulated in the cortex, 2313 in the elongation zone, 1348 in the meristematic zone, and 846 in the stele (FDR <1%; Fig. 2C). Among differentially expressed genes, more than half displayed a small absolute log_2_Fc of ≤1 (Fig. 2D; Supplementary Fig. S2). In subsequent analyses, we focused on genes with a |log_2_Fc| ≥1. This arbitrary cut-off resulted in 466 differentially expressed genes in the meristematic zone, 995 in the elongation zone, 708 in the cortex, and 345 in the stele (Fig. 2D). In the elongation zone, the highest portion (43%) of differentially expressed genes exceeded the Fc cut-off, while only a quarter of regulated genes in the cortex displayed such a high Fc (Supplementary Fig. S2). Similarly, the maximal absolute Fc was highest in the elongation zone and lowest in the cortex [Ez (340-fold) >Mz (116-fold) >St (93-fold) >Co (50-fold)].

Most differentially expressed genes (68–88%; Fig. 2D) were up-regulated. The direction of regulation was conserved among 407 (98%) of the 414 water deficit-responsive genes that were differentially expressed in more than one tissue.

Of a total of 915 water deficit-responsive genes, only 50 were consistently regulated in all tissues (48 persistently up-regulated, two persistently down-regulated), while the majority of 1501 genes were specifically regulated in a single tissue, illustrating a high degree of tissue-specific transcriptomic plasticity in response to water deficit (Fig. 2E).

### Metabolic pathway responses to water deficit

**Fig. 3.**
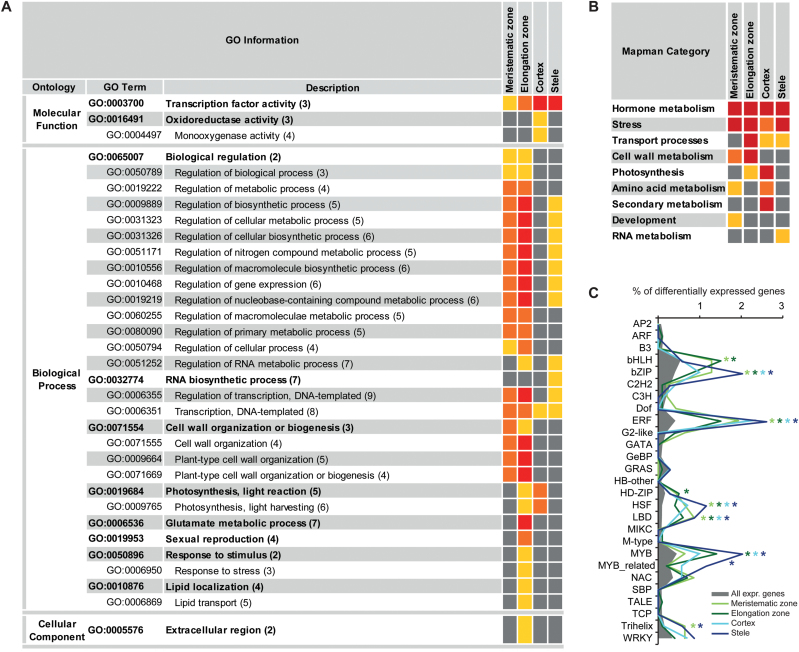
Functional categorization of differentially expressed genes. (A) Cross-comparison of enriched GO terms and (B) Mapman categories among differentially expressed genes (FDR <1%; |log_2_Fc| ≥1) in the four root tissues in response to water deficit treatment. [Numbers in parentheses indicate the GO level; different colours in the right-hand columns represent different significance levels (*q*-values in A; *P*-values in B) of the over-representation; yellow, <0.05; orange, <0.01; red, <0.001.] (C) Distribution of differentially expressed genes (FDR <1%; |log_2_Fc| ≥1) into TF families as a percentage of all differentially expressed genes (grey colour represents percentages of TFs per family among all expressed genes; only 27 TF families with >15 expressed members are shown). Asterisks indicate TF families with more differentially expressed genes than expected, χ^2^ test, *P*<0.05.

An overview of metabolic processes regulated in response to water deficit in each of the four tissues was generated with Mapman (Supplementary Fig. S3 at *JXB* online). Differentially expressed genes (FDR <1%; |log_2_Fc| ≥1) were categorized into 32 major functional Mapman categories. A variety of different biochemical pathways were affected by water deficit treatment in all tissues, including major and minor cartbohydratre (CHO) metabolism (i.e. sucrose, starch, and sugar derivate metabolism), energy generation, and lipid metabolism. A category was declared enriched if significantly more differentially expressed genes were assigned to it as expected from the distribution of all expressed genes. For all tissues, such enrichment was detected for the categories ‘hormone metabolism’, ‘stress’, and—in all but the meristematic zone—‘transport’ (Fig. 3B). In the class ‘hormone metabolism’, genes were involved in biosynthesis, degradation, and signalling processes of all plant hormones, but most in auxin, ABA, and ethylene pathways. Tissue-specific regulation was prominent, as different genes—albeit with similar functions—were affected in different tissues. Exceptions were ABA-related processes: eight genes were commonly regulated including up-regulation of the ABA biosynthesis genes *zep1*, *zep2*, and *vp14*. Most genes annotated in the category ‘stress’ belong to the subgroup ‘abiotic stress’ and encode, for example, many putative heat shock proteins (HSPs) and HSFs. In the category ‘transport’, diverse processes including transport of sugars, peptides, and amino acids were altered by water deficit. The transcriptomes of the meristematic and elongation zones further displayed an enrichment of the category ‘cell wall metabolism’. ‘Amino acid metabolism’ was significantly enriched in the meristematic zone and in the cortex. Altered pathways included, for example, down-regulation of proline degradation and up-regulation of cysteine, lysine, and tryptophan biosynthesis. In total, the cortex displayed the largest number of enriched categories, including in addition ‘secondary metabolism’ with terpenoid and phenylpropanoid metabolic processes.

### Gene Ontology categorization of water deficit-responsive genes

Further functional categorization was performed according to GO terms with agriGO. Over-represented GO categories were computed by singular enrichment analyses. In total, 33 different GO terms were over-represented, 29 in the ontology ‘biological process’, three in ‘molecular function’, and one term in ‘cellular component’ (Fig. 3A). Two categories were conserved among differentially regulated genes (FDR <1%; |log_2_Fc| ≥1) in all tissues: ‘transcription factor activity’ and ‘transcription, DNA-templated’. A comparison with the maize transcription factor database identified 199 (of 1652 expressed TFs: 10%) TFs among water deficit-responsive genes. Figure 3C displays the percentage of differentially expressed TFs within 27 major TF families. As among all expressed genes, most differentially regulated TFs belonged to bHLH, bZIP, ERF, MYB, and NAC TF families. However, different genes were regulated in the distinct tissues, with 144 (of 199) TFs being specifically affected in a single tissue. Compared with all expressed genes, bHLH, bZIP, ERF, HD-ZIP, HSF, LBD, MYB, MYB-related, and Trihelix TFs were significantly over-represented among water deficit-responsive genes in one or more tissues (Fig. 3C). In addition to gene expression regulation by TFs, further GO categories and subcategories related to regulation of transcription such as ‘RNA biosynthetic process’ were enriched among water deficit-responsive genes, in the meristematic zone, elongation zone, and stele (Fig. 3A). Additionally, many groups related to regulation of general metabolic processes were enriched in these three tissues. In the stele, no other category was over-represented. In the meristematic and elongation zone, cell wall metabolism-related processes were commonly over-represented, as already indicated by the Mapman analyses. Predominantly, up-regulated genes in this group were annotated as expansins or xyloglucan endotransglycosylases/hydrolases (XTHs). The cortex and elongation zone shared two over-represented terms, ‘photosynthesis, light reaction’ and ‘photosynthesis, light harvesting’, that included mostly genes annotated as encoding chlorophyll-binding proteins. The molecular functions ‘oxidoreductase activity’ and ‘monooxygenase activity’ corresponding to redox regulation were specifically over-represented in the cortex. The highest number of enriched GO terms was found among the responsive genes identified in the elongation zone. These included ‘glutamate metabolic process’, ‘sexual reproduction’, ‘response to stimulus/stress’, ‘lipid localization’, and the cellular component ‘extracellular region’. The last-mentioned term, however, includes many expansins that are also annotated in cell wall metabolism. This holds true for genes included in ‘sexual reproduction’ as these might be pollen allergens and/or expansins and are also annotated in both ‘cell wall organization’ and ‘extracellular region’.

### Water deficit-responsive expression changes of ‘classical maize genes’

**Fig. 4.**
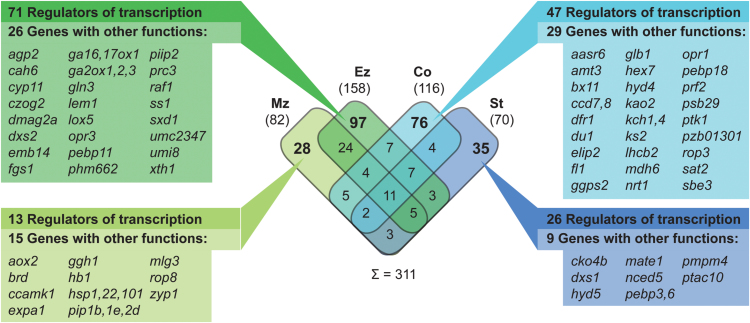
Classical maize genes among water deficit-responsive genes (FDR <1%, |log_2_Fc| ≥1) and their overlap between tissues. For tissue-specific genes, the numbers of genes with functions in regulation of gene expression (e.g. TFs) are indicated as well as genes with other functions. (For the complete list of water deficit-responsive classical genes see Supplementary Table S4 atr *JXB* online.) Mz, meristematic zone; Ez, elongation zone; Co, cortex; St, stele.

Among the 39 656 high confidence gene models of the maize FGS, 3599 are currently hand curated with experimentally confirmed functions (‘classical maize genes’; Schaeffer *et al.*, 2011; Schnable and Freeling, 2011). Of these, 311 genes were water deficit responsive (FDR <1%; |log_2_Fc| ≥1; Supplementary Table S4 at *JXB* online). A majority of 236 (76%) were tissue specifically regulated in a single tissue, a similar proportion as among all expressed genes (Fig. 4; compare Fig. 2E). More than half (>54%) of differentially expressed, classical genes encode TFs (48/82 in Mz, 97/158 in Ez, 63/116 in Co, 46/70 in St), which is a slightly higher proportion than in all expressed classical genes (1489/2989). Other water deficit-responsive, classical genes had diverse functions; many were related to stress responses, transport, or hormone metabolism. Overall, the identified water deficit-responsive classical genes reflect the functions observed for all differentially expressed genes. For instance, the aquaporin genes *pip1b*, *pip1e*, and *pip2d*, the cell division-related gene *zyp1*, and the cell elongation-related gene *expa1* were specifically regulated in the meristematic zone. Also involved in cell wall modification is *xth1* that was specifically regulated in the elongation zone upon water deficit. Furthermore, several genes involved in gibberellin (GA) metabolism (*ga16,17ox1*, *ga2ox1*, *ga2ox2*, and *ga2ox3*) were regulated in this tissue. In the cortex, the ABA-related gene *aasr6* was specifically differentially expressed, as were some genes involved in secondary metabolism (*ccd7*, *ggps2*, *hyd4*, and *ks2*) and transport of potassium (*kch1* and *kch4*). Genes solely regulated in the stele included the ion homeostasis regulator *pmpm4* (Fig. 4).

## Discussion

The first plant organ that detects a limitation of the water supply is the emerging root system. Roots represent a gradient of development, with undifferentiated cells near the tip and fully differentiated cells at the basal end. In the present study, transcriptome changes in four root tissues were surveyed to gain insight into tissue-specific water deficit responses. The meristematic zone was collected as the apical 2mm of the root and included the region in which cell division rates peak (Sacks *et al.*, 1997). Under severe water deficit conditions, fewer cells are produced and the primary root meristem is smaller than under optimal conditions (Fraser *et al.*, 1990). Sacks *et al.* (1997) detected the end of the cell division zone at 2.2mm from the root tip under control conditions and at 1.8mm under low water potentials as applied in this study. As newly formed cells start to elongate within the meristematic zone, no definite discrimination from the elongation zone can be made (Ishikawa and Evans, 1995). However, in the apical 2mm, no differences in elongation rate between control and water deficit conditions were observed (Sharp *et al.*, 1988; Fraser *et al.*, 1990; Saab *et al.*, 1992; Sacks *et al.*, 1997). Thus, the apical 2mm seems a good sample size to represent the apical meristem. Secondly, the elongation zone was sampled up to the part of the root where root hairs emerge. Root hair development is a common indicator for the region of mature cells. From the remaining part of the root, the outer cortex (containing epidermis, cortical parenchyma, and endodermis) and inner stele (including pericycle, phloem, xylem, and pith parenchyma) were sampled, corresponding to two sets of distinct cell types with very disparate physiological functions.

### Gene activity expands to more tissues upon water deficit

Per gene, the transcriptional activity (i.e. presence or absence of gene expression) was determined for each tissue–treatment combination. In contrast to analysing relative expression differences, the conversion into binary values (activity or inactivity) allows the analysis of fluctuation of expression at the level of detection (Birnbaum *et al.*, 2003). In total, 27 438 genes were active in at least one tissue–treatment combination. A similar number of 27 347 genes had been detected in a study analysing the same primary root tissues of untreated maize seedlings (Paschold *et al.*, 2014). Only 8% of the genes were specifically active in a single root tissue, which is a similar proportion to that detected by Paschold *et al.* (2014) and in maize leaves (Li *et al.*, 2010). The vast majority of genes, however, were constitutively active under both conditions in all four tissues. The remaining genes displayed specific activity patterns. They were either active in the same tissue(s) under both conditions (stable genes) or gene activity changed in response to water deficit (dynamic genes). Of the latter, many genes were active in more tissues upon water deficit than under control conditions. Furthermore, 10 genes that were inactive under control conditions but active in all tissues after water deficit treatment were identified. These genes included known stress-responsive genes such as three HSF TFs, a putative ABA signalling protein, and two putative LEA proteins. In general, functions of stable and dynamic genes were similar. However, a higher number of genes involved in stress response processes were observed among unstable genes, reflecting a highly dynamic response to water deficit.

### Cortex and elongation zone transcriptomes are most strongly affected by water deficit

Differentially expressed genes between control and water deficit treatment were identified in each tissue by pairwise comparisons. The largest effects of water deficit on gene regulation were detected in the cortex and elongation zone. The cortex displayed not only the highest number of water deficit-responsive genes but also the highest number of active genes *per se*. Similar results were obtained in analyses of *Arabidopsis* and *Brachypodium* leaves where more genes were drought responsive in mature and expansion zones (Skirycz *et al.*, 2010; Verelst *et al.*, 2012). These results indicate that differentiated tissues in general require a greater variety of gene expression levels than immature, dividing cells. The highest number of differentially expressed genes exceeding the Fc cut-off (|log_2_Fc| ≥1) was detected in the elongation zone. This strong impact of water deficit on elongating tissue reflects the uniqueness of this region as it mediates root growth responses and maintains root elongation during drought stress (see below; Sharp *et al.*, 2004).

### Water deficit responses are tissue specific and mediated by up-regulation of gene expression

Only 50 of 1915 genes were differentially expressed in all four tissues in response to water deficit. Hence, while 70% (19 163/27 438) of the active genes were constitutively active in all tissues under both conditions, <3% of water deficit-responsive genes were consistently regulated in all tissues. This indicates extensive tissue-specific dynamics of the water deficit response. In total, 78% of differentially expressed genes were specific for a single tissue. Tissue specificity of drought stress responses was also detected in individual parts of the root elongation zone (Sharp *et al.*, 2004; Zhu *et al.*, 2007; Spollen *et al.*, 2008) and distinct leaf regions of *Arabidopsis* (Skirycz *et al.*, 2011) and *Brachypodium* (Verelst *et al.*, 2012). Genes commonly regulated in more than one tissue displayed the same direction of change in the present study and in a proteome study of the maize primary root elongation zone (Zhu *et al.*, 2007). Overall, the predominant direction of expression changes was induction. This was similarly detected in other maize root zones and whole primary roots (Spollen *et al.*, 2008; Opitz *et al.*, 2014), cotton and soybean roots (Bowman *et al.*, 2013; Fan *et al.*, 2013), *Sorghum* root/shoot samples (Dugas *et al.*, 2011), maize leaf meristems (Kakumanu *et al.*, 2012), and *Arabidopsis* leaves (Skirycz *et al.*, 2010). Furthermore, gene activity analyses in this study revealed that gene activity more frequently expanded to additional tissues than decreased to fewer tissues upon water deficit. Thus, water deficit might generally trigger induction of gene expression rather than inhibition, and an increase rather than a decrease in spatial gene activity.

### Transcription factors and hormones mediate early water deficit responses in all primary root tissues

Alterations of environmental conditions trigger multiple signalling pathways that activate gene transcription and the downstream physiological adaptation. TFs play a key role in this process as main regulators of gene expression. However, expression of TFs is likewise influenced by exogenous signals. The maize FGS comprises 2231 annotated TFs. As in other plants, most of these belong to a few large multigene families such as bHLH, bZIP, ERF, and MYB (Jiang *et al.*, 2012). In this study, an over-representation of GO terms related to regulation of transcription was detected among water deficit-responsive genes in all primary root tissues. In total, ~10% of differentially expressed genes encoded TFs. About two-thirds of these were regulated in a single tissue, reflecting the high tissue-specific dynamics. Nevertheless, regulated TFs belonged to the same families such as bHLH, bZIP, ERF, and HSF that were enriched among the differentially expressed genes. Differentially expressed bHLH TFs were over-represented in the meristematic and elongation zone transcriptomes. bHLH TFs are generally up-regulated in response to drought stress and co-ordinate several processes including stomatal development, root hair formation, and hormone metabolism (reviewed in Castilhos *et al.*, 2014). Several bHLH TFs are involved in root growth control in *Arabidopsis*, for example by restricting root meristem size (Makkena and Lamb, 2013). Additionally, bHLH TFs regulate cell elongation under control of brassinosteroids (Zhang *et al.*, 2009) and therefore might be involved in growth adaptation in the root tip during water deficit. Further abiotic stress responses are mediated by bZIP TFs. Induced by ABA, they activate downstream responses such as expression of chaperones (Fujita *et al.*, 2005). In this data set, bZIP TFs were over-represented among differentially expressed genes in all tissues. However, individual genes were tissue specifically regulated. This is in agreement with the suggestion by Tang *et al.* (2012) that diverse bZIP TFs might function in distinct processes leading to stress tolerance. While bZIP TFs function via an ABA-dependent pathway, ERF TFs can integrate drought stress responses independently of ABA (Mizoi *et al.*, 2012). However, ERF TFs have also been reported to engage in ABA-mediated gene expression and to interact with TFs of other families and hormones (Xu *et al.*, 2011). In this study, ERF TFs were over-represented among regulated genes in all tissues and were the most numerous, reflecting their central role in the acquisition of stress tolerance. In comparison with the other TFs, HSF TFs have a limited influence on gene expression as they primarily regulate expression of HSPs. Most HSPs act as molecular chaperones and prevent protein unfolding and aggregation, thereby maintaining cellular protein homeostasis and cellular structures (reviewed in Wang *et al.*, 2004). Expression of HSF TFs is regulated by different stresses, including drought, and is tissue specific (Lin *et al.*, 2011; Yang *et al.*, 2014). This was also observed in the present study, where different HSF TFs were over-represented among regulated genes in all tissues. Consequently, several HSPs were up-regulated, for example the classical genes *hsp1*, *hsp22*, and *hsp101* in the meristematic zone.

Transcriptional regulation of TFs and downstream genes is co-ordinated by hormones that act as central integrators of stress adaptation signalling cascades. Thereby the balance between the different hormones determines the appropriate response to the experienced stress (Xia *et al.*, 2015). The Mapman category ‘hormone metabolism’ was over-represented among differentially expressed genes in all root tissues. Genes involved in ABA metabolism were commonly regulated in all tissues, while for all other hormone-related genes tissue specificity was pronounced. ABA is a universal stress hormone that regulates nearly 10% of the protein-coding genes under diverse abiotic stress conditions (Sreenivasulu *et al.*, 2012). The ABA biosynthesis genes *zep1*, *zep2*, and *vp14* were up-regulated in all tissues in this study. Furthermore, the *vp14* homologue *nced5* was strongly induced in the stele. The *vp14* gene had the highest Fc in the meristematic and elongation zones, reflecting the fact that ABA accumulates towards the root apex where it is involved in maintenance of elongation through prevention of excess ethylene production. ABA accumulation might also be involved in osmotic adjustment, cell wall extensibility, and ion homeostasis (reviewed in Yamaguchi and Sharp, 2010). However, root elongation is only maintained in the apical region of the elongation zone, while more basal cells cease to elongate (Sharp *et al.*, 1988). This process is co-ordinated by GA which stimulates growth of most organs during plant development. However, during stress, active GA is reduced via up-regulation of *GA2ox* genes, whose products deactivate GA (Colebrook *et al.*, 2014). Consequently, *ga2ox1*, *ga2ox2*, and *ga2ox3* were specifically up-regulated in the elongation zone in the present study, reflecting the molecular processes needed to limit cell elongation to the apical part of the elongation zone.

### Water deficit signalling induces cell protection processes in all primary root tissues

The complex interplay of different hormones and multiple TFs co-ordinates cellular responses to water deficit that include adjustments of the membrane system, modifications of the cell wall architecture, and changes in cell division. Metabolism is altered in various ways, including production of compatible solutes that are able to stabilize proteins and cellular structures and/or to maintain cell turgor by osmotic adjustment, and redox metabolism to remove excess levels of ROS and re-establish the cellular redox balance (Krasensky and Jonak, 2012). The effect of water deficit on cell membrane properties is mitigated by membrane lipid remodelling. Furthermore, lipids can act as signalling molecules during the stress response (reviewed in Okazaki and Saito, 2014). This was observed mainly in the elongation zone of the root, where the GO term ‘lipid localization/transport’ was over-represented among water deficit-responsive genes. In addition to a suitable structural barrier to the environment, ion homeostasis is crucial for physiological processes in living cells. Especially in roots, K^+^ leakage mediated by potassium channels is a common phenomenon leading to irreversible K^+^ loss under water deficit stress (Demidchik *et al.*, 2014). To prevent this, genes encoding potassium channels are down-regulated (Shabala and Pottosin, 2014); for example, *kch1* in the root cortex. Furthermore, up-regulation of *pmpm4* was specifically detected in the stele. The protein PMPM4 is involved in cation uptake and regulates the membrane potential, to maintain intracellular ion homeostasis (Fu *et al.*, 2012). Ion transport is regulated by ROS that are formed under stress (Demidchik *et al.*, 2014). ROS play a central role as signalling molecules, and spatial and temporal accumulation has a strong impact on hormone synthesis, transport, localization, and signalling (reviewed in Mittler *et al.*, 2011). However, if they reach a certain level, ROS, such as singlet oxygen, superoxide radicals, hydrogen peroxide, and hydroxyl radicals, become extremely deleterious and damage membranes, macromolecules, and nucleic acids. ROS are therefore kept under control by a scavenging system including enzymatic and non-enzymatic antioxidants (reviewed in Cruz de Carvalho, 2008). Genes encoding enzymes detoxifying ROS such as catalases, superoxide dismutases, and peroxidases were particularly over-represented in the elongation zone (GO category ‘response to stimulus/stress’) and cortex (GO category ‘oxidoreductase activity/monooxygenase activity’). Molecular chaperones that stabilize proteins and membranes and assist in protein refolding under stress conditions, especially genes encoding HSPs and LEA-type proteins, were up-regulated in all tissues and over-represented, combined in the Mapman category ‘stress’. Molecular chaperone function has also been identified for the amino acid proline. Proline can protect the integrity of different enzymes including those that act as ROS scavengers, and is further an important osmolyte. During water stress, proline biosynthesis is activated and its catabolism repressed (reviewed in Szabados and Savouré, 2010). Proline dehydrogenases that catalyse the first step of proline degradation were down-regulated in all tissues except in the stele, while transcription of several enzymes involved in proline biosynthesis was up-regulated. Furthermore, genes of the glutamate biosynthesis process were up-regulated, providing a source for proline production. In the elongation zone, this was reflected by over-representation of the GO term ‘glutamate metabolic process’ among differentially expressed genes and the up-regulation of the classic genes *fgs1* and *gln3*. In the Mapman analysis, the category ‘amino acid metabolism’ was further over-represented among regulated genes in the meristematic zone and cortex. In maize roots experiencing water deficit, proline is mainly transported to the root tip, whereby the basal region of the elongation zone might serve as a source for transport. In the elongation zone, proline might also be used for synthesis of extensins that are involved in cell wall modification during drought adaptation (Yamaguchi and Sharp, 2010).

### Cell wall loosening is induced to maintain root growth in apical root zones

Roots have the ability to elongate continuously under drought conditions. Thereby, cell elongation rates near the apex are maintained while more basal cells cease to elongate. This results in a shortened elongation zone (Sharp *et al.*, 1988). Maintenance of elongation despite reduced turgor pressure is achieved by cell wall-loosening proteins that render cell walls more extensible. Proteins with wall-loosening properties include mainly expansins and XTHs whose expression and activity increase in the root apex during drought conditions (reviewed in Wu and Cosgrove, 2000). Cell wall loosening can further be non-enzymatically enhanced by hydroxyl radicals that cause polysaccharide scission. Hydroxyl radicals are generated from hydrogen peroxide that increases in the apical elongation zone upon water deficit stress (Voothuluru and Sharp, 2012). The basal part of the elongation zone, on the other hand, ceases to elongate, and cell wall extension properties are inhibited possibly due to accumulation of lignin and cell wall-bound phenolics (Wu *et al.*, 1996; Bassani *et al.*, 2004; Fan *et al.* 2006; Spollen *et al.*, 2008). Under the experimental conditions in the present study, root growth is not visibly affected (Opitz *et al.*, 2014). Nevertheless, water deficit treatment leads to reduced turgor and cells adjust by increased expression of expansin-encoding genes to maintain local elongation rates (Yamaguchi and Sharp, 2010). Among differentially expressed genes in the apical root regions, the functional categories ‘cell wall metabolism’ and ‘cell wall biosynthesis/organization’ were over-represented, and the classical genes *exp1* and *xth1* were exclusively up-regulated in the meristematic and elongation zone, respectively. Additionally, ROS metabolism-related genes were over-represented among differentially expressed genes in the elongation zone (GO category ‘response to stimulus/stress’). Besides cell wall loosening, cell expansion requires the co-ordinated uptake of water. The transcellular water transport is dependent on the amount and activity of water channels—aquaporins—in plasma membranes (reviewed in Chaumont and Tyerman, 2014; Li *et al.*, 2014). In the present study, the 11 classical *ZmPip* genes were expressed in all root tissues, and their expression was strongest in the differentiated tissues, as similarly described by Hachez *et al.* (2006). However, following water deficit treatment, up-regulation of *pip1b*, *pip1e*, and *pip2d* was detected only in the meristematic zone, and that of *pip1f* and *pip2a* in the meristematic and elongation zones. Similar up-regulation of several *pip* genes in maize has been found in whole seedling roots (Hachez *et al.*, 2012). Such enhanced aquaporin accumulation in root tips might allow maintenance of water uptake and, together with increased cell wall loosening, ensure continuous cell division and elongation despite water deficit so that plants might reach deeper water resources.

In summary, short-term mild water deficit that does not phenotypically affect maize plants had a considerable effect on root tissue transcriptomes. Regulation of gene expression was highly tissue specific and dominated by induction. The strongest changes were detected in the elongation zone, the root region important for growth adaptation. The water deficit response was mediated by TFs and changes in hormone metabolism in all root tissues, however by distinct sets of genes. This data set provides information for future analyses needed to understand the mechanisms underlying root adaptation to water scarcity and enable targeted breeding strategies to develop more water stress-tolerant varieties.

## Supplementary data

Supplementary data are available at *JXB* online.

Figure S1. Overview of gene activity patterns and numbers of genes within these patterns.

Figure S2. Fc distribution of differentially expressed genes (FDR <1%).

Figure S3. Distribution of differentially expressed genes (FDR <1%) into major metabolic processes as visualized by Mapman.

Table S1. Overview of the sample distribution within the flow cell, biological replication, RNA-Seq output, and mapping results.

Table S2. List of activity patterns for 29 506 genes considered in a presence/absence analysis of gene expression.

Table S3. Comprehensive list of 27 386 expressed genes including normalized expression values, Fc, and *q*-values.

Table S4. Comprehensive list of expressed classical maize genes including Fc and *q*-values.

Supplementary Data
