# Supplementary material for: Extensive tissue-specific transcriptomic plasticity in maize primary roots upon water deficit
Source: J Exp Bot. 2015 Oct 13;67(4):1095–107. doi: 10.1093/jxb/erv453 (PMC4753846; doi:10.1093/jxb/erv453)
Supplement: Supplementary Data [file supp_67_4_1095__index.html]

Extensive tissue-specific transcriptomic plasticity in maize primary roots upon water deficit — Extensive tissue-specific transcriptomic plasticity in maize primary roots upon water deficit — Supplementary Data 

# Extensive tissue-specific transcriptomic plasticity in maize primary roots upon water deficit

## Supplementary Data

Data files

- Supplementary\_data.pdf - Supplementary Data
- Supplementary\_table2.xlsx - Supplementary Data
- Supplementary\_table4.xlsx - Supplementary Data
- Supplementary\_table3.xlsx - Supplementary Data
