# Supplementary material for: Extensive tissue-specific transcriptomic plasticity in maize primary roots upon water deficit
Source: J Exp Bot. 2015 Oct 13;67(4):1095–107. doi: 10.1093/jxb/erv453 (PMC4753846; doi:10.1093/jxb/erv453)
Supplement: Supplementary Data [file supp_erv453_Supplementary_data.pdf]

Exceptional tissue-specific transcriptomic plasticity in maize primary roots upon water deficit

Nina Opitz, Caroline Marcon, Anja Paschold, Waqas Ahmed Malik, Andrew Lithio, Ronny Brandt, Hans-Peter Piepho, Dan Nettleton, Frank Hochholdinger

SUPPLEMENTARY MATERIAL

Figure S1. Overview of gene activity patterns and numbers of genes within these patterns.

Figure S2. Fc distribution of differentially expressed genes (FDR <1%) in the four root tissues.

Figure S3. Distribution of differentially expressed genes (FDR <1%) into major metabolic processes as visualized by Mapman.

Table S1. Overview of the sample distribution within the flow cell, biological replication, RNA-Seq output, and mapping results.

	C \ WD	0000 (Inactive)	0001 (St)	0010 (Mz)	0011 (Mz, St)	0100 (Ez)	0101 (Ez, St)	0110 (Ez, Mz)	0111 (Ez, Mz, St)	1000 (Co)	1001 (Co, St)	1010 (Co, Mz)	1011 (Co, Mz, St)	1100 (Co, Ez)	1101 (Co, Ez, St)	1110 (Co, Ez, Mz)	1111 (Constitutive)	Σ
Inactive	**0000**	2068	75	47	11	36	4	4	1	114	26	8	10	3	5	3	10	2425
St	**0001**	67	362	2	15	3	38	0	4	3	70	3	6	1	20	0	14	608
Mz	**0010**	47	2	312	39	0	0	28	2	3	1	25	8	0	1	8	4	480
Mz, St	**0011**	3	14	14	153	0	3	0	25	0	4	3	49	0	1	0	23	292
Ez	**0100**	35	3	3	0	75	5	17	3	0	0	1	0	9	4	3	2	160
Ez, St	**0101**	3	9	0	0	1	51	0	13	0	1	0	0	0	22	0	11	111
Ez, Mz	**0110**	3	0	28	4	5	0	201	31	0	0	2	2	0	0	27	29	332
Ez, Mz, St	**0111**	1	0	2	22	1	7	13	268	0	0	1	14	0	2	5	146	482
Co	**1000**	138	13	3	1	5	0	0	1	587	152	22	12	50	38	9	21	1052
Co, St	**1001**	10	54	1	1	1	9	1	0	62	799	4	60	4	274	0	99	1379
Co, Mz	**1010**	4	1	23	1	0	0	1	1	18	3	106	38	2	0	25	14	237
Co, Mz, St	**1011**	1	4	2	20	1	0	1	2	1	45	13	398	0	10	4	297	799
Co, Ez	**1100**	6	1	0	0	13	0	4	0	16	5	2	1	147	28	24	12	259
Co, Ez, St	**1101**	2	3	0	0	1	16	0	3	6	40	2	8	12	579	1	261	934
Co, Ez, Mz	**1110**	0	1	5	0	1	0	37	3	2	2	13	6	14	4	291	114	493
Constitutive	**1111**	0	0	1	3	0	3	5	64	1	10	3	86	4	82	38	19163	19463
	Σ	2388	542	443	270	143	136	312	421	813	1158	208	698	246	1070	438	20220	29506

Color code:

- Inactive genes
- Constitutively active genes
- Stable genes
- Dynamic genes: spatial increase of gene activity after water deficit
- Dynamic genes: spatial decrease of gene activity after water deficit
- Dynamic genes: spatial change of gene activity after water deficit

Figure S1. Overview of gene activity patterns and numbers of genes within these patterns. Each cell represents a specific gene activity pattern and states the number of genes displaying this pattern. Each four digit code in first row/column represents gene activity (1) or inactivity (0) across the four tissues, in the order of cortex (Co), Elongation zone (Ez), Meristematic zone (Mz), and Stele (St). The columns indicate activity under water deficit conditions (WD), and the rows indicate activity under control conditions (C). 10,105 genes of the FGS were not considered for the analysis and could be added to the 2,068 inactive genes in the first cell (0000-0000).

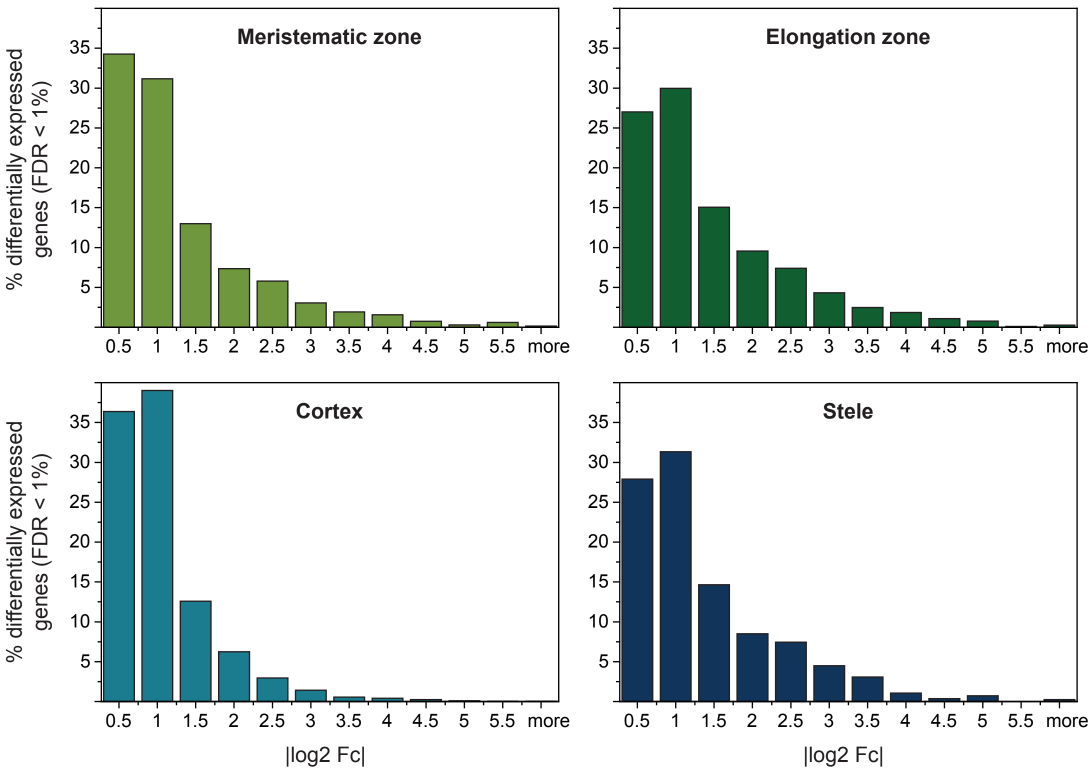

Figure S2. Fold change distribution of differentially expressed genes (FDR <1%) in the four root tissues. Bar charts display frequencies of |log2 Fc|.

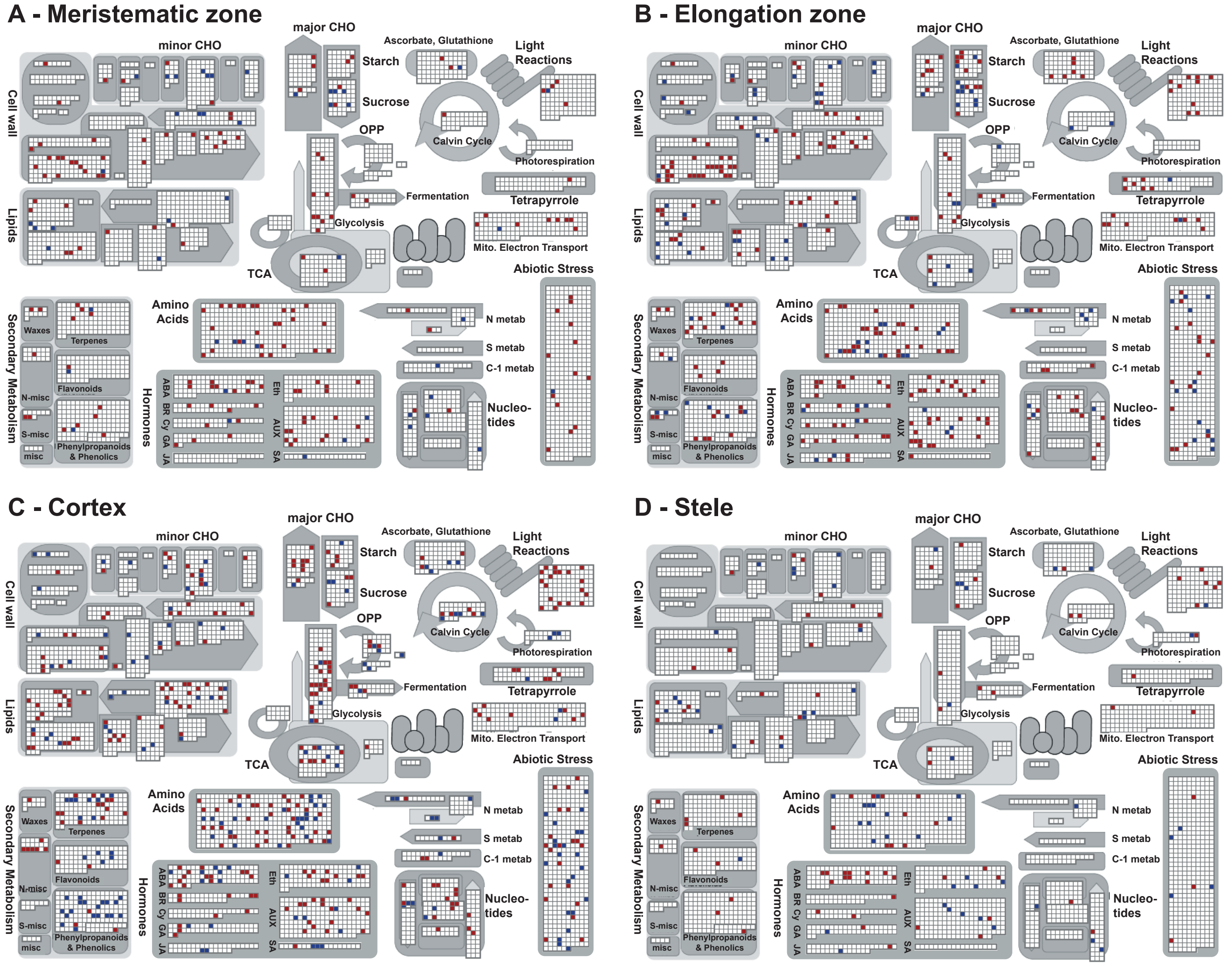

Figure S3. Distribution of differentially expressed genes (FDR <1%) into major metabolic pathways as visualized by Mapman. Genes differentially regulated in **A** meristematic zone (1,348 genes corresponding to 1,479 transcript identifiers in Mapman, visible are 203), **B** elongation zone (2,313 genes corresponding to 2,533 transcript identifiers, visible are 371), **C** cortex (2,878 genes corresponding to 3,191 transcript identifiers, visible are 467), and **D** stele (846 genes corresponding to 934 transcript identifiers, visible are 146) are idicated by colored squares in metabolic pathways. Transcripts which were up and down-regulated are represented in red and blue, respectively.

Table S1. Overview of the sample distribution within the flow cell, biological replication, RNA-Seq output, and mapping results.

Sample		Replicate	Illumina indexed adapter	Lane on Flow cell	Number of reads				
					Total - raw	Total - after trimming [a]	Uniquely mapped on genome [b]	Uniquely mapped on genome without stacks [c]	Uniquely mapped without stacks mapped on FGS [d]
Meristematic zone	control	1	AR004	2	43,935,848	43,328,679	36,615,084	16,454,296	11,774,304
		2	AR001	5	64,483,438	63,891,917	53,898,333	20,300,907	14,629,290
		3	AR011	7	65,719,035	65,208,887	55,437,932	21,462,063	15,465,324
		4	AR021	8	63,913,747	63,377,558	53,862,565	21,362,865	15,312,907
	water deficit	1	AR002	1	57,667,131	57,012,017	48,490,851	19,805,303	14,275,159
		2	AR023	6	53,699,721	53,300,735	45,610,506	19,056,195	13,800,950
		3	AR020	7	55,551,383	55,020,060	47,108,626	20,515,986	14,912,856
		4	AR022	8	47,505,365	46,906,483	39,804,432	18,259,337	13,125,390
Elongation zone	control	1	AR006	2	57,750,401	56,899,124	48,822,135	19,028,651	13,943,599
		2	AR015	4	61,403,097	60,721,818	52,362,271	18,707,962	13,712,268
		3	AR003	5	44,218,176	43,875,305	37,680,678	15,313,418	11,143,297
		4	AR006	8	41,871,561	41,415,817	34,418,106	14,998,969	10,945,694
	water deficit	1	AR019	1	52,536,520	51,826,997	44,508,242	18,030,442	13,228,438
		2	AR016	4	39,469,624	38,952,698	33,725,975	14,729,036	10,909,534
		3	AR025	6	53,039,179	52,503,793	45,304,284	18,585,944	13,725,979
		4	AR003	8	37,107,904	36,809,273	31,680,483	14,566,590	10,606,157
Cortex	control	1	AR014	2	31,066,420	30,340,990	25,786,082	10,782,034	8,103,497
		2	AR018	3	55,803,028	54,834,652	46,735,625	12,984,939	9,725,052
		3	AR004	4	51,274,131	50,151,975	42,475,851	13,053,559	9,827,509
		4	AR009	5	53,213,965	52,455,045	43,721,026	19,826,704	14,954,256
	water deficit	1	AR009	1	48,845,423	47,734,152	40,843,592	19,808,871	15,017,592
		2	AR010	3	62,472,009	60,892,714	52,204,129	20,632,083	15,561,221
		3	AR010	4	57,200,471	55,770,676	47,939,938	21,956,496	16,524,545
		4	AR018	6	59,525,450	58,611,583	49,735,045	20,229,404	15,320,460
Stele	control	1	AR007	2	26,784,079	26,372,426	22,683,174	12,979,973	9,606,068
		2	AR012	3	42,876,041	42,403,400	36,434,495	16,822,490	12,394,415
		3	AR019	5	38,915,010	38,485,887	32,922,830	15,979,628	11,817,936
		4	AR007	7	37,462,222	37,047,705	31,882,718	16,883,856	12,563,058
	water deficit	1	AR005	1	35,755,113	35,254,511	30,227,199	16,333,753	12,133,667
		2	AR013	3	49,915,834	49,400,895	41,818,382	9,069,461	6,729,209
		3	AR027	6	38,663,739	38,241,982	32,574,303	16,994,357	12,589,227
		4	AR005	7	38,724,461	38,301,754	32,863,824	17,502,500	12,977,239

[a] Quality trimming removed low quality and ambiguous nucleotides of sequence ends and adapter contamination

[b] Mapped to the reference genome of B73 (Schnable *et al.* , 2009)

[c] After removal of stacked reads i.e. redundant reads sharing the same start and end coordinate, sequencing direction, and sequence

[d] Mapped to the gene models of the "filtered gene set" (FGS) of *Zea mays* (Release 5.b)
